# Comment on “Impact of Shutdown due to COVID-19 Pandemic on Aerosol Characteristics in Kanpur, India”

**DOI:** 10.5696/2156-9614-11.29.210313

**Published:** 2021-02-25

**Authors:** Manas P. Roy

**Affiliations:** Ministry of Health and Family Welfare, New Delhi, India

The article by Shukla *et al*. is really interesting.^1^ An unprecedented restriction imposed to combat the 2019 coronavirus (Covid-19) in all spheres of life including vehicular and industrial emissions helped realize a marked reduction in air pollution everywhere. While studies from other countries have highlighted the impact of particulate matter (PM) on the prevalence of Covid-19, this impact has been explored less frequently in India.^2–4^

To estimate the effect of air pollution on Covid-19, data on air quality and Covid-19 from four districts (Delhi, Kolkata, Urban Bengaluru and Mumbai) were considered for four months (1 August to 20 November, 2020).^5–6^ The air quality index (AQI), a composite parameter based on several indicators, including PM_2.5_ and PM_10_, is used in India for summarizing the extent of air pollution. For daily and weekly newly diagnosed Covid-19 cases and deaths, data were taken for 8 August to 27 November, 2020 and compared with the AQI. A lag period of one week was maintained for estimating the association between Covid-19 cases and air pollution.

In Delhi, daily case load was strongly associated with AQI (r = 0.773), but weekly case load had an even stronger association with AQI (r = 0.869). In Kolkata, the scenario was similar (r = 0.519 and 0.707 for daily and weekly case load, respectively). It appears that the weekly relationship between Covid-19 cases and air pollution could be a better predictor than daily measurements. In Delhi and Kolkata, air pollution could explain 60% and 27% daily cases of Covid-19.

However, there was no positive association in Mumbai (r = −0.324 and −0.389 for daily and weekly case load, respectively) or urban Bengaluru (r = −0.402 and −0.525 for daily and weekly case load, respectively). In fact, these two locations indicated a negative relationship between Covid-19 cases and air pollution. In Mumbai and urban Bengaluru, however, air pollution could explain only 10% and 16% of the daily cases of Covid-19. It may be noted that the extent of air pollution is comparatively lower in these two locations.

When a similar exercise was performed for daily deaths, only Delhi had a strong positive correlation (r = 0.791). For the other three locations, air pollution was negatively related to air pollution (correlation in Kolkata = −0.150, urban Bengaluru = −0.418 and Mumbai = −0.525).

For weekly case load, the R^2^ value was 0.754 for Delhi and 0.500 for Kolkata, but 0.150 for Mumbai and 0.275 for urban Bengaluru. For weekly deaths, the R^2^ value was 0.699 for Delhi, 0.137 for Kolkata, 0.473 for Mumbai, and 0.458 for urban Bengaluru.When total deaths over the study period were considered, 51% of cases and 50% of deaths could be explained by the average AQI.

The extent of air pollution, as suggested by the analysis, was seen to influence the pandemic and related deaths only in certain locations. Future research should consider quantifying the threshold of air pollution that could potentially impact the spread of Covid-19. Pandemic mitigation strategies could consider incorporating a focus on reduction of air pollution.
